# Riboflavin Transporter Deficiency Type 2: Expanding the Phenotype of the Lebanese Founder Mutation p.Gly306Arg in the *SLC52A2* Gene

**DOI:** 10.3390/metabo15070491

**Published:** 2025-07-21

**Authors:** Jean-Marc T. Jreissati, Leonard Lawandos, Julien T. Jreissati, Pascale E. Karam

**Affiliations:** 1 Faculty of Medicine, American University of Beirut, Beirut 1107-2020, Lebanon; jtj02@mail.aub.edu (J.-M.T.J.); lel03@mail.aub.edu (L.L.); jtj03@mail.aub.edu (J.T.J.); 2 Inherited Metabolic Diseases Program, Department of Pediatrics and Adolescent Medicine, American University of Beirut Medical Center, Beirut 1107-2020, Lebanon

**Keywords:** riboflavin, neuropathy, ataxia, hearing loss, vision, *SLC52A2*, founder mutation, p.Gly306Arg, Lebanon

## Abstract

**Background**: Riboflavin transporter deficiency type 2 is an ultra-rare, yet treatable, inborn error of metabolism. This autosomal recessive disorder is caused by pathogenic mutations in the *SLC52A2* gene leading to progressive ataxia, polyneuropathy, and hearing and visual impairment. The early initiation of riboflavin therapy can prevent or mitigate the complications. To date, only 200 cases have been reported, mostly in consanguineous populations. The p.Gly306Arg founder mutation, identified in patients of Lebanese descent, is the most frequently reported worldwide. It was described in a homozygous state in a total of 21 patients. Therefore, studies characterizing the phenotypic spectrum of this mutation remain scarce. **Methods**: A retrospective review of charts of patients diagnosed with riboflavin transporter deficiency type 2 at a tertiary-care reference center in Lebanon was performed. Clinical, biochemical, and molecular profiles were analyzed and compared to reported cases in the literature. **Results**: A total of six patients from three unrelated families were diagnosed between 2018 and 2023. All patients exhibited the homozygous founder mutation, p.Gly306Arg, with variable phenotypes, even among family members. The median age of onset was 3 years. Diagnosis was achieved by exome sequencing at a median age of 5 years, as clinical and biochemical profiles were inconsistently suggestive. The response to riboflavin was variable. One patient treated with high-dose riboflavin recovered his motor function, while the others were stabilized. **Conclusions**: This study expands the current knowledge of the phenotypic spectrum associated with the p.Gly306Arg mutation in the *SLC52A2* gene. Increased awareness among physicians of the common manifestations of this rare disorder is crucial for early diagnosis and treatment. In the absence of a consistent clinical or biochemical phenotype, the use of next-generation sequencing as a first-tier diagnostic test may be considered.

## 1. Introduction

Riboflavin transporter deficiency type 2 (RTD2) is a rare treatable inborn error of metabolism, with less than 200 cases reported worldwide [1]. This autosomal recessive disorder is secondary to biallelic pathogenic mutations in the *SLC52A2* gene (OMIM # 614707), leading to an impairment of riboflavin cellular uptake. Riboflavin deficiency affects the formation of the flavin adenine dinucleotide and flavin mononucleotide, both cofactors in fatty acid β-oxidation, electron transfer flavoprotein, and complex II of the mitochondrial respiratory chain, impacting energy production [2]. RTD2 is the most common among the other known types of riboflavin transporter deficiency, type 1 and type 3, secondary to *SLC52A1* and *SLC52A3* mutations, respectively [3].

This disorder usually manifests in infancy and early childhood [4], with few cases in adulthood [5]. Clinical manifestations are variable, including sensory and motor neuropathy, ataxia, bulbar palsy, muscle weakness, optic atrophy, sensory hearing loss, and in some cases severe respiratory insufficiency. The differential diagnosis mainly includes mitochondrial disorders, neurogenetic disorders, and riboflavin transporter deficiency type 1 and type 3 [3]. Optic nerve and sensory neuropathy [6,7] seem more suggestive of RTD2; however, clinical diagnosis of this rare disorder remains challenging and is often delayed.

Biochemical investigations may reflect the impairment of acyl-CoA dehydrogenases in fatty acid β-oxidation. The plasma acylcarnitine profile could show an accumulation of short-, medium, and long-chain acylcarnitines (especially C5, C8, C10, C12, C14, C14:1, and C18), while urine organic acids may reveal elevated glutaric, ethylmalonic, and 2-hydroxyglutaric acids, resembling multiple acyl-CoA dehydrogenase deficiency [3]. All these abnormal metabolites may be inconsistently found, and both tests can be normal. Moreover, newborn screening by tandem mass spectrometry is not reliable for the detection of RTD2 [3]. Low plasma flavin levels have been reported in two cases [2].

Brain magnetic resonance imaging (MRI) and electrophysiological investigations are also non-specific and variable. Brain MRI can be completely normal, or it can show cerebellar hypotrophy and abnormal T2-weighted signal in the brainstem, cortical, and subcortical areas [3]. Electromyography–nerve conduction (EMG-NC) studies may reveal variable motor and/or sensory polyneuropathy [3].

Therefore, the diagnosis of RTD2 is based on molecular testing [3]. The first pathogenic mutation in the *SLC52A2* gene, p.Gly306Arg, was identified in 2000, in a consanguineous Lebanese family [8,9]. Since then, 33 different mutations have been reported worldwide, mostly of missense type; however, the founder mutation p.Gly306Arg remains the most common encountered allele (41.9%) [4]. Among the 200 reported patients, this mutation was identified in a homozygous state in a 21 individuals, from eight unrelated families, mostly of Lebanese ancestry [2,10].

In view of the rarity of this disorder, genotype–phenotype correlation studies are scarce [4,10,11,12], and interpretations are challenging. In addition, variable phenotypes are noted even among members of the same family, possibly due to epigenetics or modifier genes [4]. Early recognition and treatment of this neurodegenerative disorder is crucial; if left untreated, it may be fatal in childhood, mainly due to respiratory failure, with a median age of survival of 7.5 years [13]. Timely riboflavin supplementation at 10–50 mg/kg/day improves neurological manifestations, mostly ataxia, motor function, and bulbar palsy [4,14].

The aim of this study is to report the diagnosis and outcome of six new RTD2 Lebanese patients, from three unrelated families, carrying the founder mutation p.Gly306Arg in the *SLC52A2* gene. This study expands further the genotype–phenotype correlation of this ultra-rare, underdiagnosed, yet treatable inborn error of metabolism.

## 2. Materials and Methods

A retrospective review of charts of all patients diagnosed with RTD2 and followed between 1 February 2018 and 1 February 2023 at the Inherited Metabolic Diseases Program of the American University of Beirut Medical Center was performed. Clinical presentation, biochemical testing, neurological and neurophysiological investigations, molecular profiles, treatment, and outcome at last visit were recorded and analyzed. A comparative analysis with previously reported cases displaying the mutation p.Gly306Arg in the *SLC52A2* gene was also conducted.

### 2.1. Biochemical Genetics Testing

Plasma amino acids were analyzed by ion-exchange chromatography. Acylcarnitine profile determination was performed using liquid chromatography–tandem mass spectrometry at the Archimed laboratory (Vienna, Austria), following extraction from dried blood spots and derivatization by butylation to reduce interferences. Urine organic acids were analyzed by the gas chromatography–mass spectrometry of derivatized compounds at Radboud University Medical Center (Nijmegen, The Netherlands).

### 2.2. Neurological Evaluation

The neurological assessment included clinical examination, brain magnetic resonance imaging (MRI), electroencephalography (EEG), electromyography (EMG), and nerve conduction (NC) studies.

### 2.3. Ophthalmological and Audiological Assessment

Vision was evaluated by comprehensive clinical examination, electroretinography, and visual evoked potentials. Hearing assessment included pure-tone and speech audiometry, otoacoustic emissions (OAE), and auditory brainstem response (ABR) testing.

### 2.4. Genetic Testing

Exome sequencing analyses by next-generation sequencing were performed at accredited commercial genetic laboratories (Centogene GmbH, Germany, and Saint Joseph University, Lebanon). Genomic DNA was fragmented by sonication, and Illumina adapters were ligated prior to sequencing on the Illumina HiSeqX platform, achieving an average coverage depth of approximately 30×. An in-house bioinformatics pipeline was employed for base calling, primary filtering of low-quality reads and artefacts, and variant annotation. Identified variants were subjected to quality validation. Variants that met the laboratory’s internal quality control criteria—established through extensive validation—were exempted from confirmatory Sanger sequencing. Pathogenicity classification followed the American College of Medical Genetics and Genomics recommendations.

This study was approved by the Institutional Review Board of the American University of Beirut (protocol number BIO-2018-0381).

## 3. Results

### 3.1. Family 1

**Patient M1**, male, was born full term to non-consanguineous parents with no perinatal complications. He had normal psychomotor development until 3 years of age, when he developed dysarthria, fine motor difficulties (grasping and writing), unsteady gait, and frequent falls, followed by progressive deterioration of both fine and gross motor functions. He presented for evaluation at the Inherited Metabolic Diseases Program at 5 years of age. The physical examination showed muscle weakness, an inability to hold head or sit, areflexia, and an ataxic gait. The use of accessory muscles was notable upon mild physical exertion. The ophthalmological exam was normal. The basic metabolic work-up was normal, including blood muscle and liver enzymes, lactate, pyruvate, plasma amino acids, and urine organic acid chromatography. His acylcarnitine profile showed an isolated elevation of hexanoylcarnitinel. Neurological investigations including EEG and brain MRI were normal. A severe generalized sensory neuropathy was documented by EMG and NC studies. Audiometry revealed bilateral sensorineural hearing loss.

Finally, exome sequencing revealed a homozygous pathogenic mutation, p.(Gly306Arg) in the *SLC52A* gene, diagnostic of RTD2. The patient was supplemented with riboflavin at a dose of 50 mg/kg/day. Three months later, his gross motor skills began to improve progressively. At last follow-up, at 8 years of age, he was able to walk again unsupported; however, he still had a mild ataxic gait with early signs of lumbar scoliosis.

**Patient M2**, a male sibling of patient M1, was detected at the age of 3 years 8 months by genetic screening after his brother’s diagnosis, while still asymptomatic. He was found homozygous for the p.(Gly306Arg) in the *SLC52A* gene. He had intact vision and hearing and age-appropriate language development. Biochemical investigations revealed a normal acylcarnitine profile and urine organic acid chromatography. He was treated with riboflavin supplementation at a dose of 25 mg/kg/day. At last follow-up, at 6 years 8 months of age, he was still asymptomatic.

### 3.2. Family 2

**Patient M3**, a male, born to consanguineous parents, with normal perinatal history and psychomotor development, was diagnosed at 2 years of age with mild lumbar scoliosis. At 4 years of age, he displayed a progressive ataxic gait associated with frequent falls. A pediatric neurology work-up including brain MRI, spine MRI, EMG-NC and EEG, was normal. No further investigations were conducted. He was seen at the Inherited Metabolic Diseases Program at 6 years of age for worsening ataxic gait. His family history was positive for parental consanguinity and one older sibling, patient M4, who died at 7 years of age from an undiagnosed “neurodegenerative disease”. The neurological exam was positive for decreased deep tendon reflexes and proximal muscle weakness. The patient had normal vision and hearing. The metabolic work-up revealed an abnormal acylcarnitine profile with elevated C4, C6, and C8 suggestive of possible fatty acid oxidation. Exome sequencing revealed a homozygous pathogenic mutation p.(Gly306Arg) in the *SLC52A* gene, diagnostic of RTD2. The patient was started on riboflavin supplementation at 25 mg/kg/day. Six months later, there was no change in the ataxic gait. The riboflavin dose was increased to 50 mg/kg/day; however, the parents stopped the treatment, and the patient was lost to follow-up for 4 years and 6 months. He was seen again at 11 years of age, with hearing loss, vision impairment, and worsening of ataxia. The parents reported during this period a rapid progression of the lumbar scoliosis, for which he underwent 12 surgeries. Riboflavin supplementation was restarted at 50 mg/kg/day. At last follow-up, at 11 years 6 months, he was stable with no further deterioration in his gait.

**Patient M4,** the brother of patient M3, was reportedly normal up to 4 years of age; then, he progressively suffered from isolated muscle weakness affecting mostly the proximal limbs. Biochemical investigations (blood creatine phosphokinase, arterial lactate, pyruvate, plasma amino acids, and urine organic acid chromatography) were normal. Brain MRI and EEG were negative. An EMG-NC study was consistent with polyneuropathy. He was suspected of having a neurodegenerative disease. At 7 years of age, he died undiagnosed due to respiratory failure triggered by an intercurrent viral infection. He was retrospectively diagnosed with RTD2 based on his clinical profile and family history.

### 3.3. Family 3

**Patient M5**, male, presented to the Inherited Metabolic Diseases Program at 7 years of age for progressive ataxia and a history of hearing loss. He was born full term to consanguineous parents. At 2 years of age, he presented with an abnormal gait and hearing difficulties. EMG-NC was suggestive of axonal polyneuropathy with normal brain and spine MRI. Visual reinforced audiometry showed bilateral sensorineural hearing loss. At 4 years and 6 months of age, he had a right cochlear implantation performed for the sensorineural hearing loss. His physical exam was positive for proximal muscle weakness, ataxia, spine thoracic scoliosis, and areflexia. The ophthalmological exam was normal. The metabolic work-up was negative except for elevated C16 by the plasma acylcarnitine profile. He was diagnosed with RTD2 by exome sequencing, which showed a biallelic homozygous pathogenic mutation p.(Gly306Arg) in the *SLC52A* gene. Riboflavin supplementation was initiated at 25 mg/kg/day. Three months later, he was stable. The patient was lost to subsequent follow-up.

**Patient M5**, a 4-year-old female and sibling of patient M4, was identified by genetic screening after her brother’s diagnosis. She developed a mild ataxic gait at 3 years of age. Physical examination was normal, except for ataxia and a mild left thoracolumbar curvature, suggestive of scoliosis. The hearing test and ophthalmological examination were normal. Her acylcarnitine profile was also normal. Treatment with riboflavin was started at 25 mg/kg/day. At last follow-up, three months later, she was still stable.

## 4. Discussion

This study describes six newly identified patients with RTD2 from three unrelated Lebanese families all harboring the same missense homozygous mutation, p.Gly306Arg, in the *SLC52A2* gene. These findings broaden the phenotypic spectrum previously reported in eight families in the existing literature [2,9,10,12,15,16]. This missense mutation has been previously suggested as a founder variant in our population [10,12], which is consistent with the high consanguinity rate of up to 35.5% [17]. Of the 21 cases homozygous for the p.Gly306Arg mutation, 17 were of Lebanese ancestry. Among the ten patients with the mutation in a compound heterozygous state, eight were of English origin [10], one was Caucasian of unspecified ancestry [18], and one was Polish [19].

The pathogenicity of the p.Gly306Arg variant in the *SLC52A2* gene has been functionally characterized in several in vitro studies, demonstrating impaired riboflavin transporter activity. Johnson et al. first described this homozygous mutation in a Lebanese family. In vitro experiments showed that it markedly reduced cellular uptake of riboflavin and disrupted membrane localization, likely due to protein misfolding or mistrafficking [9]. Subsequently, Foley et al. performed detailed uptake assays and a membrane fraction analysis in HEK293 cells, revealing that the p.Gly306Arg variant leads to a moderate but significant reduction in riboflavin transport (25–30% relative to the wild type) despite preserved mRNA levels [10]. Recently, Zhang et al. demonstrated that the loss-of-function effect caused by this mutation results in a marked decrease in riboflavin transport activity and impairs oxidative phosphorylation, which clinically manifests as neurodegeneration resembling mitochondrial energy disorders [13].

In this study, a total of 27 patients were analyzed. The majority (88%) were of Lebanese origin (Table 1). Although RTD2 is an autosomal recessive disorder, a male predominance was observed in 64% of the patients. The median age of clinical onset was 3 years, in line with other reported cases [3]. The median age at diagnosis and treatment in this study was 5 years, which is relatively earlier than the median age of 10 years previously reported in a cohort of 21 patients [4]. In fact, the location of the p.Gly306Arg variant in the C-terminal region in the SLC52A2 gene is usually associated with earlier symptom onset [4]. This may also be related to the improved availability of genetic testing by next-generation sequencing compared to earlier cases diagnosed nearly two decades ago.

A variability in age of onset and phenotypic expression was observed among siblings within the same family. In Family 1, patient M1 was already symptomatic at 3 years of age, whereas his brother, M2, was still asymptomatic at 3 years 8 months and remained symptom-free following riboflavin supplementation. Ataxia was the presenting symptom at 2 years of age in patient M3, while his sibling, M4, did not exhibit signs of ataxia up to his death at 7 years of age. Furthermore, although patient M5 presented at 2 years of age with muscle weakness along with hearing loss and polyneuropathy, his sister, M6, had only isolated mild ataxia at 3 years of age. The phenotypic heterogeneity of RTD2 patients highlights a multifactorial interaction beyond simple genotype–phenotype correlations. Potential phenotypic modifiers such as epigenetic factors, co-existing genetic variants, or environmental influences may explain the intra-familial variability observed. Zhao et al. demonstrated that individuals homozygous for the variant p.Gly306Arg commonly exhibited high rates of ataxia, while those with compound heterozygous variants primarily developed respiratory insufficiency [4]. Manole et al. [20] proposed that neurons co-expressing multiple riboflavin transporters may compensate for transporter dysfunction, whereas neurons relying on a single defective transporter are more likely associated with severe mitochondrial alterations. Furthermore, Qiao et al. [21] provided clear evidence that fluoride exposure directly altered the expression of *SLC52A2* and *SLC52A3*, disrupted mitochondrial enzyme activity, and induced interleukin-17A-driven inflammation, thereby demonstrating that environmental factors can modulate riboflavin transporter expression and mitochondrial function, potentially influencing disease onset or severity. In addition, infections or immune triggers sometimes precede clinical onset in genetically predisposed individuals, implying a contributory role of environmental or autoimmune factors in unmasking the disease [22].

Ataxia was the dominant clinical feature, in concordance with Zhao et al. [4]. The underlying cause of this gait disturbance could be attributed to dysfunctional proprioception secondary to progressive neuronopathy [6]. In addition to ataxia, the most common phenotypic manifestations were sensorineural hearing loss and polyneuropathy, followed by muscle weakness. Visual impairment was encountered in less than half of the patients (44%). Although muscle weakness increases the risk of scoliosis, only 33% developed progressive scoliosis but without respiratory compromise. Gayathri et al. recently highlighted that the abnormal curvature of the vertebral column [23] leads to a mechanical compression of the thoracic cavity, thus reducing lung volume and limiting diaphragmatic excursion. Therefore, annual spinal radiography is recommended [14] for early scoliosis detection and proactive management, including orthotics and physiotherapy, to mitigate long-term pulmonary sequelae [3]. None of the patients showed signs of bulbar palsy, in parallel with previous reports [3].

Auditory neuropathy is another well-established hallmark of RTD2. Sensorineural hearing loss results from vestibulocochlear nerve degeneration and can progress to complete bilateral deafness [3]. Menezes et al. [24] showed that hearing loss initially presented with preserved outer hair cell function alongside abnormal auditory brainstem responses, followed by a rapid progression to profound deafness within two years. Hearing loss may be reversed if riboflavin therapy is initiated within 12 months of symptom onset [14]. While the usage of hearing aids is not useful, cochlear implants might be beneficial in unresponsive patients after 12 months of riboflavin therapy [24]. Patient M5 received a cochlear implant almost 3 years before he was diagnosed with RTD2, although he was displaying suggestive symptoms of this disorder.

These findings highlight the diagnostic challenges and the need for early intervention in RTD2. Increased awareness among physicians of the common manifestations of this rare disorder is crucial for early diagnosis and treatment. In patients presenting with early-onset sensorineural hearing loss with or without suggestive neurological signs, such as ataxia, peripheral neuropathy, or muscle weakness, a detailed audiological assessment should be conducted. This should include auditory brainstem response testing, even in the presence of preserved outer hair cell function. In addition, comprehensive genetic testing should also be considered for the early diagnostic confirmation of this actionable and preventable disorder.

The biochemical diagnosis of RTD2 patients remains challenging, as it can be inconsistently abnormal and non-specific. Plasma acylcarnitine profiles by tandem mass spectrometry performed in 19 out of 27 patients were abnormal in 44%, suggesting mitochondrial fatty acid oxidation dysfunction.

Response to riboflavin therapy was also variable among patients (Table 2). In this study, despite diagnosis and therapy initiation before 5 years of age, three patients remained stable, whereas the patient diagnosed at 5 years of age and treated with riboflavin at 50 mg/kg/day showed improvement in motor function and was able to walk again. One asymptomatic patient treated with riboflavin at 25 mg/kg/day did not develop any symptoms after 3 years of follow-up. In fact, there is considerable variability in riboflavin dosing regimens reported in the literature, and no universally accepted standardized protocol has been established to date. This may be attributed to the heterogeneity in patient age, symptom onset, clinical severity, and the rate of disease progression [14]. Although riboflavin doses ranging from 10 to 50 mg/kg/day have been shown to improve or stabilize neurological and auditory function, the optimal dosage remains unclear. The response to riboflavin therapy may also be influenced by the mutation type and timing of therapy initiation [14]. Late-diagnosed patients, after the onset of clinical symptoms, would warrant not only riboflavin supplementation but also expensive multidisciplinary care involving neurologists, audiologists, otolaryngologists, and orthopedic specialists. Therefore, the initiation of riboflavin supplementation is currently recommended, even before genetic testing results, given the effectiveness and safety of this treatment [25].

**Table 1:** Phenotypic expression of the Lebanese founder mutation p.Gly306Arg in the *SLC52A2* gene causing riboflavin transporter deficiency type 2.

Family ^ref^	Origin	Sex	Patient	Clinical Phenotype (from Onset to Last Evaluation)						
				Onset	Ataxia	NP *	MW	SNHL	Scoliosis	Visual Impairment
**F1 ^[23]^**	Israeli Arab	M	P1	3 y	+		-	-	-	+
	Israeli Arab	F	P2	3 y	+		+	+	-	+
**F2 ^[16]^**	Lebanese	F	P3	3.5 y	+	+	+	+	-	-
	Lebanese	M	P4	2.5 y	+	+	-	+	-	+
	Lebanese	M	P5	1.5 y	+	-	+	+	-	-
	Lebanese	M	P6	2 m	-	-	-	+	-	-
**F3 ^[10]^**	Lebanese	F	P7	8 y	+	+	-	+	-	-
	Lebanese	F	P8	3 y	+	+	+	+	-	+
**F4 ^[10]^**	Scottish	F	P9	1.5 y	+	+	+	+	-	+
	Lebanese	M	P10	3 y	+	+	+	+	-	+
	Lebanese	M	P11	5 y	+	+	+	+	-	+
	Lebanese	M	P12	3 y	+	+	+	+	-	+
**F5 ^[10]^**	Lebanese	M	P13	3 y	-	+	+	+	-	-
**F6 ^[12]^**	Lebanese	M	P14	1.5 y	+	+	+	+	+	-
	Lebanese	M	P15	2.5 y	+	+	+	+	+	+
	Lebanese	F	P16	3.5 y	+	+	+	+	+	+
	Lebanese	F	P17	3 y	+	+	+	+	+	+
	Lebanese	M	P18	1.5 y	+	+	-	-	-	-
**F7 ^[20]^**	NR	NR	P19	1 m	+	+	+	+	-	-
	NR	NR	P20	3 y	+	-	+	+	-	-
**F8 ^[15]^**	Lebanese	F	P21	2.5 y	+	+	-	-	+	-
F9 *	Lebanese	M	P22/M1	3 y	+	+	+	+	+	-
	Lebanese	M	P23/M2	AS	-	-	-	-	-	-
F10 *	Lebanese	M	P24/M3	2 y	+	+	+	+	+	+
	Lebanese	M	P25/M4	4 y	-	+	+	-	-	-
F11 *	Lebanese	M	P26/M5	2 y	+	+	+	+	+	-
	Lebanese	F	P27/M6	3 y	+	-	-	-	+	-
***Total***	Lebanese **(22/25)** *88%*	9/25 F *(36%)* **12/25** M *(64%)*	27	Median 3 y	**4/27** *85%*	**20/27** *74%*	**8/27** *70%*	**21/27** *77%*	**(9/27)** *33%*	**(12/27)** *44%*

M: male, F: female, NR: not reported, y: year, m: months, NP: polyneuropathy, MW: muscle weakness, SNHL: sensorineural hearing loss. *: this study.

**Table 2:** Biochemical profiles, riboflavin therapy, and outcomes of patients with the Lebanese founder mutation p.Gly306Arg in the *SLC52A2* gene associated with riboflavin transporter deficiency type 2.

Family ^ref^	Origin	Sex	Patient	Biochemical Phenotype *Acylcarnitine profile*	Riboflavin Treatment	Treatment Onset Age	Age at Last Follow-Up	Outcome
F1 ^**[23]**^	Israeli Arab	M	P1				27 y	Motor delay
	Israeli Arab	F	P2				17 y	Regression
F2 ^**[16]**^	Lebanese	F	P3				5 y	Motor delay
	Lebanese	M	P4				9 y 6 m	Stable
	Lebanese	M	P5				5 y 6 m	Stable
	Lebanese	M	P6				1 y	Motor, speech delay
F3 ^**[10]**^	Lebanese	F	P7	↑ C4, C5, C6, C10, C;10:1	23 mg/kg/d	10 y	10 y 7 m	Improved hearing, motor
	Lebanese	F	P8	↑ C5, C6, C8, C10, C:12	26 mg/kg/d	9 y	9 y 7 m	Stable
F4 ^**[10]**^	Scottish	F	P9	↑ C4–C6, C2, C10-C18	50 mg/kg/d	10 y 6 m	11 y 6 m	Improved hearing,
	Lebanese	M	P10	Normal	1000 mg/d	16 y	16 y 7 m	Improved motor
	Lebanese	M	P11	Normal	1000 mg/d	21 y	21 y 7 m	Stable
	Lebanese	M	P12	Normal	1000 mg/d	16 y	167 m	Stable
F5 ^**[10]**^	Lebanese	M	P13	Normal	10 mg/kg/d	6 y6 m	7 y 7 m	Improved motor
F6 ^**[12]**^	Lebanese	M	P14	↑ C6, C8, C10, C10:1	10–15 mg/kg/d	19 y	19 y 3 m	Improved motor
	Lebanese	M	P15				22 y	Death
	Lebanese	F	P16	Normal	10–15 mg/kg/d	17 y	17 y 3 m	Improved motor
	Lebanese	F	P17				20 y	Stable
	Lebanese	M	P18	↑ C6, C8, C10, C10:1	10–15 mg/kg/d	4 y	4 y 3 m	Improved motor
F7 ^**[20]**^	NR	NR	P19	-	20–26 mg/kg/d	10 y	11 y	Improved hearing
	NR	NR	P20	-	20–26 mg/kg/d	9 y	10 y	Improved motor
F8 ^**[15]**^	Lebanese	F	P21	↑ C16	10.5 mg/kg/d	9 y	15 y	Improved ataxia
F9 *	Lebanese	M	P22/M1	↑C6	50	5 y	8 y	Improved motor
	Lebanese	M	P23/M2	Normal	25	3 y 8 m	6 y 8 m	Normal
F10 *	Lebanese	M	P24/M3	↑ C4, C6, C8	50	6 y/11 y **^#^**	11 y 6 m	Stable
	Lebanese	M	P25/M4				7 y	Death
F11 *	Lebanese	M	P26/M5	↑ C16	25	7 y	7 y 3 m	Stable
	Lebanese	F	P27/M6	normal	25	4 y	4 y 3 m	Stable
***Total***	**Lebanese** **(22/25) 88%**	**F: (9/25) 36%** **M: (12/25) 64%**	**27**	**(7/16)** ***44%***	**Range: 10–50** **mg/kg/d**	**Median: 10 y** **Range: 4 y–19 y**	**Median: 12 y 6 m** **Range: 1 y–27 y**	

M: male, F: female, NR: not reported, y: year, m: months, NP: polyneuropathy, MW: muscle weakness, SNHL: sensorineural hearing loss, mg/kg/d: mg/kg/day, #: treatment for 6 months at 6 years of age then stopped and resumed at 11 years, ↑: increased, *: this study.

In view of the non-specificity of RTD2 clinical presentation, the inconsistency of first-tier diagnostic tests, and the absence of specific diagnostic metabolites, early diagnosis is challenging, requiring the “exome/genome first” or the multi-gene panel approach [26]. RTD2 is being more frequently diagnosed in recent years due to the advances and availability of genetic testing, mainly next-generation sequencing. Recent studies suggest that incorporating exome or genome sequencing early in the diagnostic pathway of rare diseases increases diagnostic yield and results in substantial cost savings [27,28]. Further cost–benefit analyses may be required to compare the financial implications of using next-generation sequencing as a first-tier test with the long-term expenses incurred for managing RTD2 complications through a multidisciplinary approach.

Even though this condition does not fully meet the criteria for newborn screening [22], incorporating such founder mutations into newborn screening programs needs to also be considered, particularly in highly consanguineous populations.

Early identification of this disorder could mitigate the clinical manifestations [14] or prevent them, with possibly a better quality of life for patients treated early.

This study has some limitations. The retrospective design may have caused selection and information biases, as data were extracted from existing medical records. Moreover, the small number of patients, due to the rarity of this disorder, reduces the statistical power and may not reflect the complete phenotypic spectrum, thereby limiting the generalizability of the findings. Furthermore, the variability in follow-up duration among patients limits the ability to draw firm conclusions on long-term treatment efficacy and outcome. Larger prospective studies are needed to address these limitations.

## 5. Conclusions

This study enhances the current phenotypic knowledge of the most common mutation causing RTD2. Increased physician awareness of the manifestations of this disorder is critical to ensure timely diagnosis and effective intervention. In the absence of a consistent clinical or biochemical phenotype for this rare but treatable inborn error of metabolism, the use of next-generation sequencing as a first-tier diagnostic test may be discussed.

## Data Availability

The original contributions presented in this study are included in the article. Further inquiries can be directed to the corresponding author.

## Abbreviations

The following abbreviations are used in this manuscript:

RTD2	Riboflavin transporter deficiency type 2
MRI	Magnetic resonance imaging
EMG-NC	Electromyography–nerve conduction
EEG	Electroencephalography
OAE	Otoacoustic emissions
ABR	Auditory brainstem response
M	Male
F	Female
NR	Not reported
y	Year
m	Months
NP	Polyneuropathy
MW	Muscle weakness
SNHL	Sensorineural hearing loss
mg/kg/d	mg/kg/day
